# Discovery of the MELK–Nucleostemin Axis in Glioblastoma: Implications for p53 Regulation and Tumor Progression

**DOI:** 10.4014/jmb.2510.10047

**Published:** 2026-01-21

**Authors:** Songyi Baek, Hyojin Jeon, Jae-Su Moon, Young Eun Kim, Dukjin Kang, Sunghwan Kim, Kwang-Rok Kim, Kyung-Sun Heo

**Affiliations:** 1 Center for Rare Disease Therapeutic Technology, Therapeutics & Biotechnology Division, Korea Research Institute of Chemical Technology (KRICT), Daejeon, Republic of Korea; 2 College of Pharmacy, Chungnam National University, Daejeon, Republic of Korea; 3 Group of Biometrology, Korea Research Institute of Standards and Science, Daejeon, Republic of Korea; 4 Mass Spectrometry Convergence Research Institute, Kyungpook National University, Daegu, Republic of Korea

**Keywords:** Glioblastoma, MELK-NS axis, p53 Signaling, Proteasomal degradation

## Abstract

Glioblastoma multiforme (GBM) is the most common and aggressive primary brain tumor, classified as a World Health Organization (WHO) grade IV astrocytoma. Despite multimodal therapies, the prognosis of patients with GBM remains poor, with a median survival of only 12–16 months. The highly invasive nature and therapeutic resistance of GBM underscore the need to identify novel molecular targets. Maternal embryonic leucine zipper kinase (MELK), a serine/threonine kinase of the Snf1/AMPK family, is highly expressed in GBM and regulates cell proliferation, cell cycle progression, and stemness; however, its downstream mechanisms are unclear. Nucleostemin (NS, GNL3) is a nucleolar GTP-binding protein involved in cell proliferation and p53 regulation; however, its regulation in GBM has not been fully elucidated. In this study, we identified NS as a novel MELK substrate in glioblastoma U87MG cells. MELK directly interacts with and phosphorylates NS, promoting its proteasomal degradation. MELK overexpression decreased NS expression, leading to enhanced p53 activation and G1 cell cycle arrest. Conversely, MELK knockdown restored NS stability and attenuated p53 activation. These findings define a previously unrecognized MELK–NS–p53 signaling axis that links kinase activity to the regulation of the cell cycle. Our fundings provide mechanistic insights into glioblastoma pathogenesis and suggest that targeting the MELK–NS pathway may be a potential therapeutic strategy for high-grade gliomas.

## Introduction

Glioblastoma multiforme (GBM), also known as glioblastoma, is a grade IV astrocytoma. Glioblastoma begins in the brain and is the most common and aggressive cancer [1]. GBM tumor cells have highly heterogeneous, invasive, necrotic, and chemotherapy- and radiation-resistant natures [2]. These highly invasive and resistant apoptosis properties of GBM are the major problem for an effective cancer treatment [3]. Cancer therapies include chemotherapy, radiation, radiosurgery, corticosteroids, antiangiogenic therapy, surgery, and experimental approaches such as gene transfer [4]. Treatment generally involves surgery, followed by chemotherapy and radiation therapy [5]. Despite decades of various clinical research, the survivability of GBM patients remains abysmal, with an average median survival of only 12–16 months [6]. Because of its severely aggressive nature and poor therapeutic properties, understanding the etiology of GBM is an urgent problem and a great interest [7]. Above all, we studied human glioblastoma U87MG cells, a commonly studied grade IV cell line. It has epithelial morphology and adherent properties, and was obtained from 44 years 44-year-old cancer patient [8].

Serine/threonine kinases are promising proteins for targeted cancer therapy. In particular, MELK has been described as a potential anticancer target in diverse tumor types [9]. MELK is a member of the snf1/AMPK family of protein serine/threonine kinases that is maximally active during mitosis [10]. MELK is involved in diverse cell progresses, such as cell proliferation, cell cycle, apoptosis, self-renewal, RNA processing, and embryonic development [11]. In previous studies, MELK is significantly highly expressed in human cancers of colon, breast, ovaries, pancreas, prostate, and brain (glioblastoma multiforme) compared with normal cells [9]. An evaluated MELK expression is shown to be more aggressive forms of astrocytoma, breast cancer, melanoma, and glioblastoma [12]. These suggest that MELK plays a critical role in the proliferation and invasion of cancer cells, and MELK may be a compelling molecular target for the treatment of high-grade brain tumors. MELK can regulate and interact with various proteins by phosphorylation [13]. In previous studies, MELK phosphorylates and binds to the zinc finger-like protein 9 (ZPR9), and also interacts with nuclear inhibitor of protein phosphatase-1 (NIPP1), PDK1, CDC25B, p53, and Smad protein [13-16]. However, knowledge of the exact function and mechanism of MELK remains unclear. Consequently, these new findings of MELK substrate provided some insights for various cellular processes and mechanisms by MELK activity.

NS, also known as guanine nucleotide-binding protein-like 3 (GNL3), regulates the cell cycle and affects cell differentiation, and its levels decrease during differentiation [17, 18]. The expression of NS reduces before cell cycle departure upon differentiation of the stem cells, suggesting that this protein may play an important role in stem cell proliferation [17]. NS is concentrated in the nucleolus of most stem cells and many tumor cells and has been implicated in cell cycle progression via its ability to regulate p53. Both overexpression and reduction of NS activate p53 by restraining MDM2 activity [19, 20]. When NS overexpressed, it stabilizes p53 and induced cell cycle arrest by directly binding to MDM2 and inhibiting MDM2-induced p53 suppression [19, 21]. In contrast, NS knockdown using siRNA induced p53 activation by improving the binding of ribosomal proteins L5 and L11 to MDM2 [21]. However, the exact function of NS remains unclear.

Here, we report the identification of NS as a novel MELK substrate in glioblastoma U87MG cells. We show interactions between MELK and NS proteins and the phosphorylation of NS by MELK. In addition, our results indicate that the reduction of NS by MELK overexpression affects the activation of p53 and induction of G1 arrest.

## Material and Method

### Cell Lines and Plasmids, and RNA Interference

U87MG human malignant glioma cells were cultured in Dulbecco’s modified Eagle’s medium (DMEM) supplemented with 10% fetal bovine serum (FBS), 50 U/ml penicillin, and 0.1 mg/ml streptomycin at 37°C in a humidified incubator with 5% CO_2_. U87MG cells (ATCC HTB-14, USA), originally derived from a patient with stage IV glioblastoma, exhibit an epithelial morphology and were maintained according to standard procedures.

To generate a MELK-inducible stable cell line (U87MG-melk), the Tet-On 3G doxycycline-inducible expression system (Clontech, 631168, Japan) was used. Cells were co-transfected with pCMV-Tet3G and pTRE2-hygro vectors, which confer neomycin and hygromycin resistance, respectively. Stable clones were selected using 200 μg/ml neomycin and 100 μg/ml hygromycin. MELK cDNA amplified from U87MG RNA was inserted into the pTRE2-hygro vector. Doxycycline treatment activated the Tet-On 3G transactivator produced from pCMV-Tet3G, enabling dose-dependent induction of MELK expression [22, 23]. The U87MG-melk cells were maintained in medium containing the same antibiotic concentrations. Additional expression constructs, including pCMV6-Entry–Myc-DDK–MELK, pCMV6-Entry–MELK, pCMV6-Entry–Myc-DDK–NS, and pCMV6-AC–NS, were purchased from OriGene [24].

Protein–protein interaction between MELK and NS was examined using the CoralHue Fluo-Chase system (MBL, AM-1100M, Japan), which employs split monomeric Kusabira-Green fluorescent fragments (mKG-N and mKG-C) to visualize protein interactions in living cells. Full-length MELK and NS cDNAs were cloned into mKG expression vectors, and U87MG cells were transfected with the resulting constructs. Reconstitution of mKG fluorescence was monitored 24–48 h after transfection, indicating physical proximity of MELK and NS [25, 26].

RNA interference–mediated knockdown of endogenous MELK and NS was performed using siRNAs purchased from Santa Cruz Biotechnology. U87MG cells were transfected with MELK siRNA or NS siRNA using Lipofectamine RNAiMAX (Invitrogen, USA) according to the manufacturer’s instructions, and cells were harvested 24 hours post-transfection for subsequent analyses [27].

### Transient Transfection and Western Blot Analysis

For transient transfection, U87MG cells were plated in 100-mm culture dishes and transfected with plasmid DNA using Lipofectamine LTX and Plus reagent (Invitrogen). Cells were seeded to reach 60 to 70 percent confluence on the day of transfection. A total of 400 pmol siRNA was mixed with Lipofectamine RNAiMAX and diluted to a final concentration of 40 pmol/mL for cell treatment. The amount of RNAiMAX used followed the manufacturer’s instructions. The siRNA–RNAiMAX complexes were incubated for five minutes at room temperature and then added dropwise to the culture medium. Cells were incubated for up to 48 h after transfection prior to further analyses. Cells were harvested and lysed using the PRO-PREP protein extraction kit (Intron, 17081, Korea). Briefly, cells were washed twice with PBS, scraped, and incubated in lysis buffer (2× pellet volume) for 15 min on ice. Protein lysates were mixed with 4× Laemmli sample buffer (Bio-Rad), and 20 μg of total protein per sample was separated by SDS–PAGE (Bio-Rad) and transferred to PVDF membranes (Bio-Rad, USA). Membranes were blocked in 1× blocking buffer for 1 h and subsequently incubated with primary antibodies overnight at 4°C. After washing, membranes were incubated with horseradish peroxidase–conjugated secondary antibodies, and protein signals were detected using enhanced chemiluminescence (Amersham, UK).

### Immunoprecipitation Assay

Co-immunoprecipitation assays were performed following transient transfection of U87MG cells. Cells were seeded to reach 60 to 70 percent confluence in 6-well plates and transfected with DDK-MELK and DDK-NS expression vectors using Lipofectamine 3000 (Thermo Fisher Scientific, L3000001, USA) according to the manufacturer’s instructions. For each well, 1.0 μg of DDK-MELK and 1.0 μg of DDK-NS plasmid DNA were used, maintaining a total DNA amount of 2 μg. For immunoprecipitation (IP) analyses, cells were harvested 24 h post-transfection and lysed in IP lysis buffer (Thermo Fisher Scientific, 87787) containing 25 mM Tris-HCl (pH 7.4), 150 mM NaCl, 1% NP-40, 1 mM EDTA, and 5% glycerol. For proteasome inhibition studies, cells were treated with 20 μM MG132 for the indicated times before lysis, and lysates were supplemented with Halt protease and phosphatase inhibitor cocktail (Thermo Fisher Scientific, 78440). Cells in 100-mm dishes were washed with PBS, scraped, and incubated in 500 μl of lysis buffer for 15 min on ice. Lysates were clarified by centrifugation at 13,000 rpm for 15 min, and the supernatants were incubated with primary antibodies for 2 h at 4°C. Immune complexes were captured by adding Dynabeads Protein (Invitrogen, 10001D) and rotating overnight at 4°C. Bead-bound complexes were eluted with 4× Laemmli sample buffer (Bio-Rad, 1610747) and analyzed by Western blotting.

### Antibodies

The following antibodies were used: anti-MELK (Abcam, ab108529, UK), anti-GAPDH (Santa Cruz, sc-47724, USA), anti-Cdc25B (Santa Cruz, sc-65504), anti-phospho-Cdc25B (Santa Cruz, sc-130184), anti-NS (Santa Cruz, sc-166460), Anti-DDK (BioLegend, 637319, USA), anti-pan-phospho-serine/threonine (LSBio, LS-C185887, USA), β-actin (Sigma, A5441, USA), anti-ubiquitin (BioLegend, 838703, USA), and anti-phospho-Erk1/2 (Santa Cruz, sc-136521).

## Cell Proliferation Assay

Cell proliferation was assessed using the CCK-8 assay (Dojindo Laboratories, CK04-11). U87MG cells were transfected with siRNA for 24 h and subsequently seeded into 96-well plates. For MELK-inducible experiments, U87MG-melk stable cells were treated with 200 or 400 ng/ml doxycycline for 24 h before seeding into 96-well plates.

Cell viability was measured using the WST-8–based CCK-8 reagent, which generates a water-soluble formazan dye upon reduction by cellular dehydrogenases. The amount of formazan produced correlates directly with the number of viable cells. After cell seeding, 10 μl of CCK-8 solution was added to each well, followed by incubation for 3 h at 37°C. Absorbance was measured at 450 nm using a microplate reader.

## Invasion and Migration Assay

Cell migration and invasion were evaluated using the CytoSelect 96-well Cell Migration and Invasion Assay Kit (Cell Biolabs, CBI-CBA-106-C, USA) according to the manufacturer’s instructions. The assay employs polycarbonate membrane inserts (8-μm pore size) for migration assays and basement membrane–coated inserts for invasion assays. U87MG-melk cells were suspended in serum-free medium at a concentration of 1 × 10^5^ cells/ml. To the lower wells (feeder tray), 150 μl of medium containing FBS with or without 200 ng/ml doxycycline to induce MELK expression was added. The membrane chamber was then placed onto the feeder tray, and 100 μl of the cell suspension was added to each insert. Plates were incubated for 24 h at 37°C to allow cells to migrate or invade through the membrane. After incubation, cells that migrated or invaded to the underside of the membrane were detached using cell detachment buffer, lysed, and quantified using CyQUANT GR fluorescent dye. Fluorescence was measured at 480/520 nm using a microplate reader.

## *In vitro* Kinase Assay

NS phosphorylation by MELK was assessed using an *in vitro* kinase assay. Recombinant wild-type MELK protein and glutathione S-transferase (GST)–tagged wild-type NS protein were used. GST–NS (1 μg) was incubated with recombinant MELK (0, 30, or 300 nM) in kinase buffer containing 50 mM HEPES (pH 7.0), 0.05% GSA, 5 mM MgCl_2_, and 1 mM DTT, in the presence or absence of ATP (0 or 100 μM). Reactions were carried out at 25°C for 24 h. Reaction products were resolved by SDS–PAGE and analyzed by Western blotting using a phospho–serine/threonine antibody (LSBio, LS-C185887) to detect NS phosphorylation.

## Results

### MELK Is Related to Cell Proliferation, Migration, and Invasion in Glioblastoma U87MG Cells

**Fig. 1:**
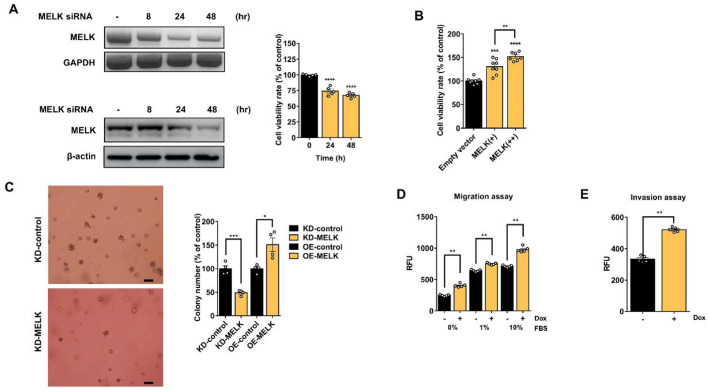
Effect of MELK expression change on survival, invasion, and migration of glioblastoma U87MG cells. (**A**) MELK knockdown reduces cell proliferation. U87MG cells were transfected with MELK siRNA and harvested after 8, 24, or 48 h. MELK mRNA and protein levels were analyzed by RT-PCR and Western blotting (left), and proliferation was measured by CCK-8 assay (right). MELK expression was significantly reduced following siRNA treatment, resulting in a marked decrease in cell proliferation. (**B**) MELK overexpression enhances cell proliferation. U87MG cells were transfected with a MELK expression vector and incubated for 24 h. Proliferation increased in a dose-dependent manner with MELK expression. “MELK (+)” indicates low-level MELK induction with 200 ng/ml doxycycline, and “MELK (++)” represents higher induction with increased doxycycline exposure. Empty vector cells served as the control. (**C**) MELK promotes anchorage-independent growth. Soft agar colony formation was significantly decreased by MELK siRNA compared with control siRNA. Conversely, colony numbers were increased upon MELK overexpression (scale bar, 100 μm). The x-axis compares two experimental groups: MELK knockdown (control vs. MELK siRNA) and MELK overexpression (empty vector vs. MELK expression vector). (**D** and **E**) MELK overexpression enhances migration and invasion. U87MG-melk stable cells were treated with doxycycline to induce MELK expression and seeded into transwell migration (**D**) or invasion (**E**) assays. Doxycycline-induced MELK overexpression significantly increased both migratory and invasive capabilities. All data are means ± SEM. Statistical significance: *p value < 0.05, **p value < 0.01, ***p value < 0.001, ****p value < 0.0001. Graphs were analyzed using GraphPad Prism 6 software. KD, knockdown; OE, overexpression.

To investigate the effect of MELK on the proliferation of glioblastoma U87MG cells, we first performed transfection of MELK siRNA in a time-dependent manner. As shown in Fig. 1A (left panel), transfection with siMELK markedly reduced both MELK mRNA and protein levels at 48 h compared with the control. Consistent with this reduction, siMELK significantly suppressed U87MG cell proliferation, as determined by CCK-8 assay at 48 h post-transfection [28]. In contrast, transient overexpression of MELK induced a dose-dependent increase in cell growth (Fig. 1C) [29]. These results demonstrate that MELK expression positively regulates the proliferative capacity of U87MG glioblastoma cells.

To further investigate the mechanism of MELK action, we assessed the phosphorylation status of several proteins previously reported as MELK substrates, including Cdc25B, p53, Smad2, and Smad3. U87MG cells transfected with siMELK were subjected to Western blot analysis; however, MELK knockdown did not result in appreciable changes in the phosphorylation of these proteins (Fig. S1) [14]. These findings suggest that MELK may act on alternative downstream targets in U87MG cells.

To modulate MELK expression in U87MG cells, we generated a stable inducible cell line, U87MG-MELK, by co-transfecting MELK expression vectors containing a Tet-On regulatory system. In this system, doxycycline treatment activates the vectors, thereby initiating MELK transcription and protein expression. Upon the addition of doxycycline to the culture medium, MELK expression was robustly induced in U87MG-MELK cells. Treatment with 200 ng/ml doxycycline induced MELK expression in a time-dependent manner (Fig. 1C). Consistent with previous findings [29]. doxycycline-treated U87MG-MELK cells exhibited a significant, dose-dependent increase in cell proliferation. Moreover, induction of MELK expression with 200 ng/ml doxycycline enhanced both cell migration and invasion (Fig. 1D and 1E) [30, 31]. These findings indicate that MELK expression is associated with enhanced cell proliferation, migration, and invasion, suggesting that MELK may contribute to the regulation of cell growth and metastatic potential in U87MG glioblastoma cells.

### MELK Interacts with Nucleostemin in U87MG Cells

**Fig. 2:**
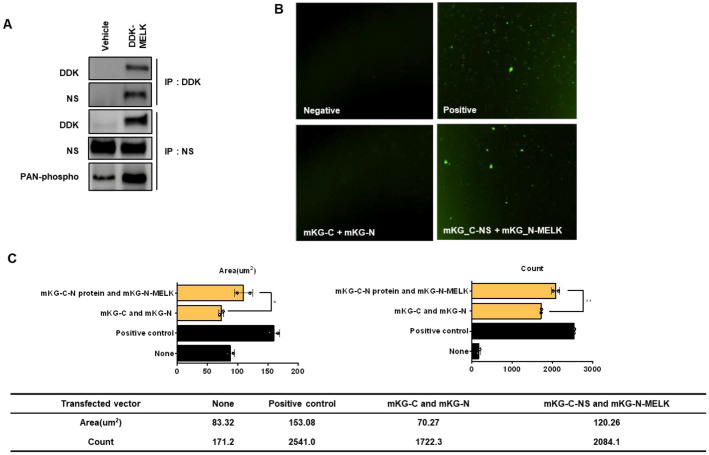
Interaction between MELK and NS on U87MG cells. (**A**) Interaction between MELK and NS was examined by immunoprecipitation in U87MG cells. Cells were transfected with an empty vector or a DDK-tagged MELK expression vector for 24 h. Whole-cell lysates were subjected to immunoprecipitation using anti-DDK or anti-NS antibodies, followed by Western blot analysis with NS, DDK, and pan–phospho-serine/threonine antibodies to assess binding and phosphorylation. MELK was detected in complex with NS, and NS phosphorylation was increased in the presence of exogenous MELK. (**B**) Fluorescence-based visualization of MELK–NS interaction using the CoralHue Fluo-Chase system. Representative fluorescent images of U87MG cells expressing negative controls (upper left), positive control constructs (upper right), mKG-C and mKG-N fragments alone (lower left), and mKG-C–NS together with mKG-N–MELK (lower right) are shown. Co-expression of mKG-C–NS and mKG-N–MELK resulted in strong reconstitution of mKG fluorescence, indicating direct protein–protein interaction. (**C**) Quantitative analysis of fluorescence intensity in the CoralHue Fluo-Chase assay. Data are presented as means ± SEM. Statistical significance was determined using GraphPad Prism 6 software (*p < 0.05, **p < 0.01).

There was no MELK-dependent change in the expression of proteins known as MELK substrates in U87MG cells (Fig. S1). To investigate the MELK substrate in U87MG cells, we first performed a transfection experiment using a DDK-tagged MELK expression vector and IP analysis using DDK antibody. The eluted IP sample was analyzed using LC/MS to determine which substrate interacted with MELK. LC/MS analysis revealed that NS is a significant target of MELK (data not shown). Furthermore, we verified binding between MELK and NS by immunoprecipitation experiments using DDK antibody from cell lysates. Endogenous MELK was immunoprecipitated from cell lysates using an anti-MELK antibody, and the binding between MELK and NS was confirmed in U87MG cells (Fig. 2A) [32]. To determine whether phosphorylation of NS changes by MELK expression, we performed transfection using a DDK-tagged MELK expression vector and IP analysis using an NS antibody. Consequently, we verified interaction between MELK and NS in U87MG cells, and NS phosphorylation showed an increase following MELK induction (Fig. 2B) [32]. To further characterize the MELK-NS interaction, we tried to protein-protein interaction analysis using the Coral Hue Fluo-chase kit. The mKG-NS and mKG-MELK expression vectors were constructed by cloning the full-length NS and MELK into the mKG vector, as a reporter protein, respectively. The U87MG cells were co-transfected with mKG-NS and mKG-MELK vectors for 48 h, and fluorescence signals were observed using SpectraMax i3 device (Molecular Devices, i3X ,USA). As shown in Fig. 2C, co-transfections of both positive vectors and cloning vectors of mKG-NS and mKG-MELK resulted in relatively detected fluorescent signals as contrasted with both non-transfected and mKG empty vector-transfected cells [33, 34]. Fig. S2 shows the Coral Hue Fluo-chase results for the additional group. Several fluorescence expression cotransfected cloning vectors inserted MELK and NS relatively increased in U87MG cells. Especially, the cells cotransfected with both mKG-C-NS and mKG-N-MELK observed a remarkable fluorescence signal. These results suggest that MELK interacts with NS and regulates phosphorylation of NS in U87MG cells.

### MELK Phosphorylates NS *In Vitro*

**Fig. 3:**
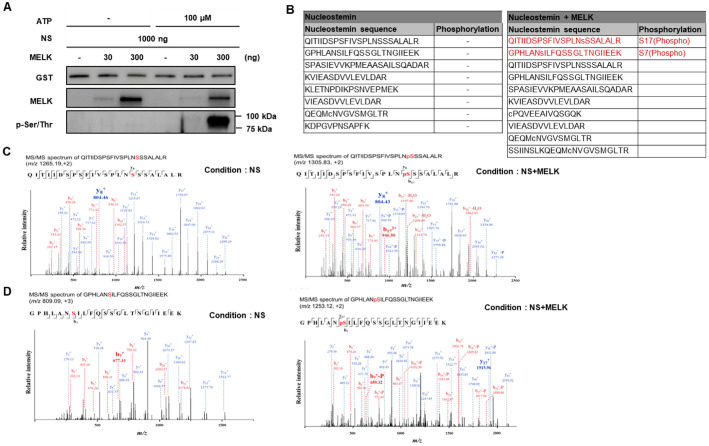
Phosphorylation of NS, as MELK substrate, using *in vitro* kinase assay and LC-MS/MS analysis. (**A**) GST-tagged NS (1 μg) was incubated with recombinant MELK protein (0, 30, or 300 ng) for 24 h at 25°C in the presence or absence of 100 μM ATP. Reaction products were analyzed by Western blotting using antibodies against NS, MELK, and phospho–serine/threonine. NS phosphorylation increased in a concentration-dependent manner upon addition of recombinant MELK. (**B-D**) In vitro kinase reaction samples generated under the same conditions were subjected to LC–MS/MS analysis. Mass spectrometry confirmed MELK-dependent phosphorylation of NS, identifying two phosphorylated peptide sequences: QITIIDSPSFIVSPLNSSSALALR and GPHLANSILFQSSGLTNGIIEEK (**B**). Extracted ion chromatograms and MS/MS spectra demonstrated clear phospho-site peaks for each peptide (**C, D**), validating that NS is directly phosphorylated by MELK *in vitro*.

To confirm NS phosphorylation by MELK *in vitro*, we performed *in vitro* kinase assay using recombinant MELK and NS proteins. We verified phosphorylation change of NS via Western blot assay, after NS recombinant protein was reacted with or without MELK recombinant protein at 25°C for 24 h in the presence or absence of 100 μM APT. As shown in Fig. 3A, phosphorylation of NS was fairly increased by the amount of MELK in a dose-dependent manner when compared with the absence of MELK control [35]. To examine thoroughly whether MELK phosphorylates NS as a substrate, we analyzed samples reacted with NS and MELK recombinant protein under the foregoing conditions. As a result of LC/MS analysis, we observed two phosphorylated NS sequences, QITIIDSPSFIVSPLNpSSSALALR and GPHLANpSILFQSSGLTNGIIEEK, as MELK substrates (Fig. 3B). As MS/MS spectrum data, we confirmed an elevated mass peak of two NS sequences caused by phosphorylation (Fig. 3C and 3D) [36]. The phosphorylation of recombinant NS proteins in the presence of MELK was observed, indicating that NS can act as substrates of MELK.

### NS Is Regulated by MELK through Proteasomal Degradation

**Fig. 4:**
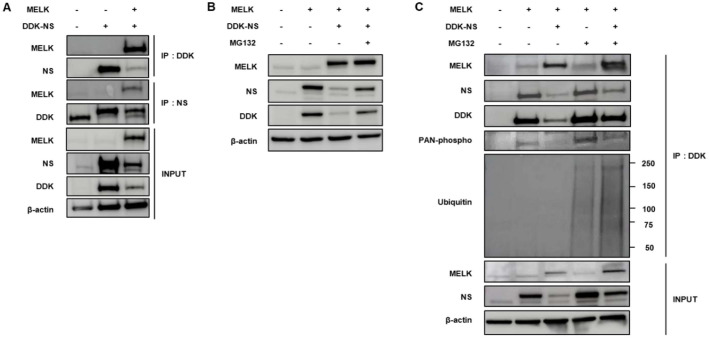
Degradation and ubiquitylation of NS by MELK overexpression on U87MG cells. (**A**) NS protein levels were reduced following MELK overexpression. U87MG cells were co-transfected with DDK-tagged NS and either MELK or an empty vector. DDK-NS was immunoprecipitated using a DDK antibody, and MELK–NS complexes were analyzed by Western blotting with MELK and NS antibodies. Exogenous MELK expression markedly decreased NS protein levels. (**B**) Proteasome-dependent degradation of NS induced by MELK. U87MG cells were transfected with DDK-NS alone or together with MELK and subsequently treated with DMSO or 20 μM MG132 for 24 h. Western blot analysis showed that MG132 treatment rescued the reduction of NS caused by MELK overexpression, indicating proteasome-mediated degradation. (**C**) MELK-driven ubiquitination of NS. U87MG cells were co-transfected with DDK-NS and MELK or vector control in the presence or absence of MG132. DDK-NS was immunoprecipitated, and ubiquitination was assessed by Western blotting using an antiubiquitin antibody. Upregulation of MELK increased NS ubiquitination, correlating with enhanced degradation of NS.

We observed that when the cells were co-transfected with DDK-tagged NS and MELK vector, the NS expression was dramatically reduced (Fig. 4A). We thought NS, as a substrate for MELK, may be degraded by MELK in U87MG cells. To confirm whether NS expression is decreased by upregulation of MELK expression, the cells were treated with 20 μM MG132, a proteasomal degradation inhibitor, after transfection for 24 h. As shown in Fig. 4B, proteasomal degradation inhibitor MG132 blocked the downregulation of NS by overexpression of MELK, and NS was rescued from MELK-mediated reductions in U87MG cells [37, 38].

Also, ubiquitination, phosphorylation, and expression of NS were significantly increased by treatment with MG132 after co-transfection with MELK and NS vectors (Fig. 4C). When the cells were cultured with MG132 for 4, 8, and 24 h after transfection, ubiquitin-mediated degradation of NS was rescued, and phosphorylation was also significantly increased in the presence of MG132 in a time-dependent manner (Fig. S3). In other words, NS expression is regulated by phosphorylation via MELK and degraded by ubiquitination; therefore NS expression was stabilized in the presence of MG132 in U87MG cells [39].

### Expression Changes of MELK Lead to Alteration of NS Expression MELK

**Fig. 5:**
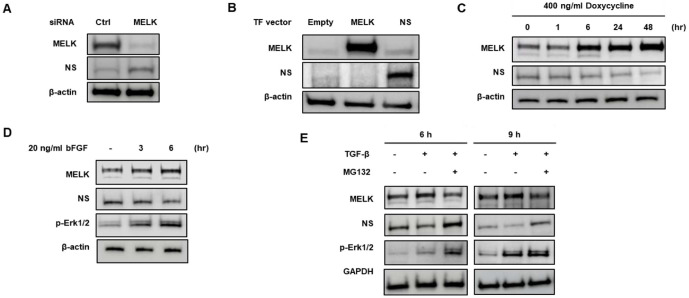
Effect on NS expression by change of MELK protein level in U87MG cells. (**A**) MELK knockdown increases NS expression. U87MG cells were transfected with MELK-specific siRNA or control scrambled siRNA for 48 h, and whole-cell lysates were analyzed by Western blotting with MELK and NS antibodies. Reduction of MELK expression resulted in a corresponding increase in NS protein levels. (**B**) MELK overexpression decreases NS expression. U87MG cells were transfected with plasmids expressing MELK or NS, and cell lysates were subjected to Western blot analysis. Forced MELK expression led to a marked reduction in NS protein levels. (**C**) Inducible MELK overexpression represses NS in U87MG-melk stable cells. U87MG-melk cells were treated with 400 ng/ml doxycycline for 1, 6, 24, or 48 h to induce MELK expression. A time-dependent increase in MELK expression resulted in progressive repression of NS. (**D, E**) Regulation of NS expression by MELK stimulation via bFGF or TGF-β. U87MG cells were serum-starved for 24 h and treated with 20 ng/ml bFGF (**D**) or 24 ng/ml TGF-β (**E**) in the absence or presence of MG132 (20 μM). Both bFGF and TGF-β stimulation reduced NS expression in a time-dependent manner, consistent with MELK activation. NS repression was rescued by MG132 treatment, indicating proteasome-mediated degradation.

We investigated whether MELK reduction using siRNA altered the expression of NS. U87MG cells were transfected with control siRNA or MELK siRNA for 48 h. NS expression was inevitably upregulated following the suppression of MELK expression (Fig. 5A) [28, 32]. A unilateral upregulation of MELK by transfection, on the other hand, leaded to suppress NS expression in U87MG cells (Fig. 5B). NS expression was reduced by doxycycline-induced MELK overexpression in U87MG-melk stable cells alike (Fig. 5C)[13]. TGF-β and bFGF were included in our analysis because these ligands are major upstream regulators of glioblastoma stem-like signaling and have been shown to activate pathways that converge on MELK. Prior studies demonstrate that both factors enhance MELK expression and kinase activity in neural stem-like glioma cells; therefore, we hypothesized that these stimuli may regulate NS stability through MELK activation. In addition, we sought to investigate the MELK–NS–p53 relationship because both MELK and NS independently modulate p53 via MDM2-dependent mechanisms. Given that NS was identified as a MELK substrate, determining whether MELK-mediated NS degradation contributes to p53 inactivation was essential for defining the functional significance of the MELK–NS axis. Therefore, we next examined whether NS expression is influenced by MELK stimuli: TGF-ß (24 ng/ml) or bFGF (20 ng/ml). We verified phosphorylation of Erk using Western blot assay, because if cells are treated with bFGF or TGF-ß, activation of Erk will occur. Exposure to bFGF and TGF-ß resulted in a significant decline of NS expression (Fig. 5D and 5E). As shown in Fig. 5E, NS expression reduced by TGF-ß-induced MELK upregulation was stabilized by treatment with MG132. These suggest that regulation of MELK expression by transfection or stimuli, such as TGF- ß and bFGF, has an important effect on NS expression in U87MG cells.

### NS Depletion–Induced MELK Overexpression Promotes MDM2–p53 Interaction and p53 Degradation

**Fig. 6:**
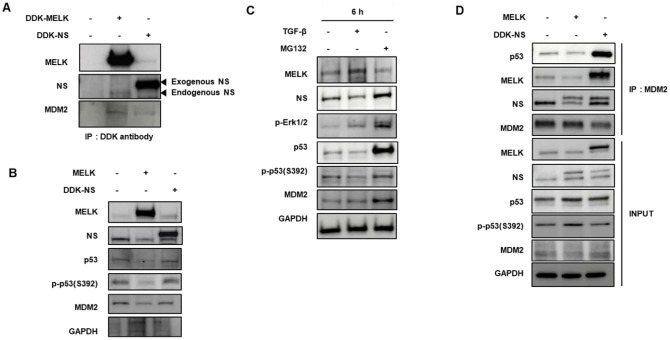
Induction of MDM2-p53 interaction and MDM2-mediated p53 degradation by NS depletion induced MELK overexpression. (**A**) Interaction of MELK and NS with MDM2. U87MG cells were transfected with the indicated expression vectors, and cell lysates were subjected to immunoprecipitation using an anti-DDK antibody. Western blot analysis revealed that both MELK and NS formed complexes with MDM2 in U87MG cells. (**B**) MELK suppresses p53 activation, whereas NS stabilizes and activates p53. U87MG cells were transfected with MELK or NS expression vectors and analyzed by Western blotting. MELK overexpression reduced p53 phosphorylation and protein stability, while NS overexpression enhanced p53 phosphorylation and accumulation. (**C**) TGF-β–induced MELK expression inhibits p53 phosphorylation and stability. U87MG cells were serum-starved for 24 h and treated with 24 ng/ ml TGF-β for 6 hours in the presence or absence of MG132. TGF-β–mediated MELK induction decreased p53 expression and phosphorylation, whereas MG132 treatment rescued p53 levels. (**D**) MELK enhances the interaction between p53 and MDM2. U87MG cells were transfected with DDK-NS alone or co-transfected with DDK-NS and MELK, followed by immunoprecipitation using an anti-MDM2 antibody. NS expression reduced MDM2–p53 binding, whereas co-expression of MELK significantly increased the interaction between p53 and MDM2.

To better understand how MELK and NS influence p53 signaling, we first sought to clarify the molecular relationship among these factors, given that both MELK and NS have been independently implicated in cell-cycle regulation, stemness, and tumor progression, yet their coordinated impact on p53 activity remains poorly defined. In particular, because MDM2 serves as a central E3 ligase controlling p53 stability, we hypothesized that MELK and NS may regulate p53 at least in part through modulating MDM2–p53 interactions. To test this, we examined whether MELK and NS physically associate with MDM2 in U87MG cells. Immunoprecipitation assays revealed that both MELK and NS interacted with MDM2, suggesting their potential involvement in modulating MDM2 function (Fig. 6A). Functional assays demonstrated that MELK overexpression led to repression of p53 activation and destabilization of the protein, whereas NS overexpression enhanced p53 phosphorylation and stabilization (Fig. 6B). Consistent with these findings, stimulation with TGF-β, which induced MELK expression, markedly suppressed p53 expression and phosphorylation. Treatment with MG132 restored p53 stability, indicating that the observed reduction was mediated through proteasomal degradation (Fig. 6C). Moreover, inducible NS expression decreased the binding affinity between MDM2 and p53, while co-expression of MELK with NS significantly enhanced this interaction(Fig. 6D)[40]. Collectively, these findings indicate that MELK functions as a negative regulator of p53 by strengthening its association with MDM2, thereby promoting MDM2-mediated p53 degradation, whereas NS exerts an opposing, p53-stabilizing effect.

### MELK Overexpression Attenuates NS-Mediated Apoptosis in U87MG Cells

**Fig. 7:**
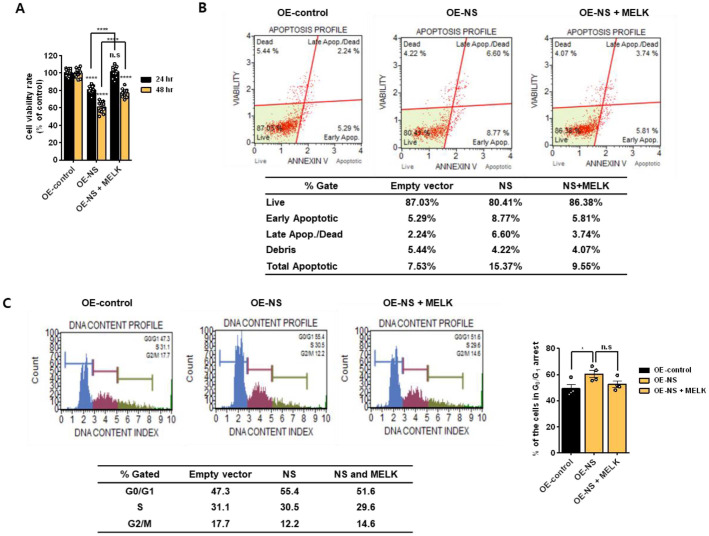
Effect of MELK overexpression for NS-mediated apoptosis U87MG cells. (**A**) Cell proliferation assay of U87MG cells transfected with an NS overexpression vector in the presence or absence of MELK overexpression. Proliferation was measured at 24 and 48 h following transfection. (**B**) Annexin V apoptosis analysis of U87MG cells transfected with empty vector control (left), NS overexpression vector (middle), or NS overexpression vector together with MELK (right). NS overexpression increased apoptotic cell populations, whereas MELK co-expression reduced NS-induced apoptosis. (**C**) Muse cell-cycle analysis of U87MG cells transfected with empty vector control (left), NS overexpression (middle), or NS overexpression with MELK (right). NS overexpression induced G0/G1 cell-cycle arrest, and MELK co-expression attenuated this effect. All data are presented as means ± SEM. Statistical significance was determined using GraphPad Prism 6 software (*p < 0.05, ****p < 0.0001). OE, overexpression.

As shown in Fig. 6, MELK facilitated MDM2-mediated p53 degradation, whereas NS exerted an opposing effect by stabilizing p53. To further determine the functional consequence of this regulatory interplay between MELK and NS, we next examined their roles in cell proliferation and apoptosis. To assess the functional interplay between MELK and NS in regulating cell survival, proliferation assays were performed after transfecting U87MG cells with NS in the presence or absence of MELK overexpression. NS overexpression significantly reduced cell proliferation at both 24 and 48 h, indicating its pro-apoptotic effect. However, concomitant MELK overexpression alleviated this growth suppression, suggesting that MELK counteracts the anti-proliferative activity of NS (Fig. 7A). Consistent with these findings, Annexin V analysis demonstrated that NS overexpression markedly increased the apoptotic cell populations compared with the vector control. In contrast, co-expression of MELK with NS diminished the extent of apoptosis, further supporting the antagonistic role of MELK in NS-induced apoptotic signaling (Fig. 7B). Muse cell cycle analysis revealed that NS overexpression caused G0/G1 phase arrest, suggesting that NS inhibits cell cycle progression at the checkpoint before DNA replication (Fig. 7C). Collectively, these results indicate that MELK suppresses NS-mediated apoptosis, thereby contributing to the enhanced survival of U87MG cells.

## Discussion

In this study, we identified NS as a novel substrate of MELK and demonstrated that MELK directly interacts with and phosphorylates NS, promoting its ubiquitin-mediated proteasomal degradation in glioblastoma U87MG cells. These findings expand the known functions of MELK substrates beyond CDC25B, p53, and Smad proteins, and uncover a previously unrecognized signaling axis linking MELK activity to NS regulation [13, 29, 36, 41-43].

Functionally, MELK-mediated NS destabilization provides a mechanistic connection between kinase signaling and cell-cycle control. NS is known to regulate proliferation and p53 activity in stem and tumor cells, and its expression is enriched in highly proliferative glioma populations [19]. Our data suggest that MELK-driven degradation of NS may contribute to glioblastoma cell proliferation, migration, and invasion. This mechanism is consistent with previous evidence implicating MELK in maintaining stem-like features and promoting aggressive tumor behavior. Thus, the MELK–NS axis represents a biologically relevant pathway that may underlie the oncogenic effects of MELK in glioblastoma.

The identification of NS as a phosphorylation substrate of MELK also provides potential insight into p53 regulation. Given the established role of NS in modulating p53 stability, MELK-induced NS degradation may offer an indirect route through which MELK influences p53 activity and cell-cycle arrest. This adds a new dimension to MELK’s function within tumor signaling networks and highlights the broader impact of its kinase activity on nucleolar protein homeostasis [44-46]. Although the magnitude of apoptosis and cell-cycle changes observed in our functional assays was relatively modest, such effects are consistent with upstream regulatory events. Because GBM survival pathways are highly redundant, subtle yet statistically significant alterations can still represent biologically meaningful modulation. Therefore, the MELK–NS axis is likely to act as one of several contributing regulators of apoptotic and cell-cycle control rather than a singular dominant pathway.

From a therapeutic perspective, the MELK–NS axis may represent a clinically actionable vulnerability. MELK inhibitors are under clinical investigation for various malignancies, including glioblastoma, and our findings suggest that modulation of NS stability could be one mechanism contributing to their antitumor activity. Further integration of patient-derived datasets may help clarify the relevance of MELK and NS expression patterns in glioblastoma progression.

OTSSP167, a potent ATP-competitive MELK inhibitor, has demonstrated robust antitumor activity in multiple preclinical cancer models, including glioblastoma. Previous studies have reported that OTSSP167 suppresses proliferation, invasion, stem-like phenotypes, and tumor-initiating capacity in glioblastoma and glioma stem cells. Given our finding that MELK drives NS degradation, MELK inhibition may stabilize NS, thereby restoring NS-mediated p53 activation and cell-cycle arrest. These observations raise the possibility that NS protein levels could serve as a pharmacodynamic biomarker for assessing on-target efficacy of MELK inhibitors such as OTSSP167. Incorporating NS expression as a downstream readout may enhance patient stratification and improve the translational application of MELK-targeted therapies.

Despite these insights, several questions remain. The precise molecular mechanism by which MELK-mediated phosphorylation promotes NS ubiquitination is still unclear, including the identity of the responsible E3 ligase. Rescue experiments are needed to determine whether the cellular effects of MELK depend directly on NS destabilization. In addition, validation across multiple glioblastoma models and *in vivo* systems will be essential to establish the generalizability and physiological relevance of this pathway. Future studies evaluating the MELK–NS pathway in additional GBM cell lines with diverse genetic backgrounds, as well as *in vivo* tumor models, will be critical to determine whether the signaling axis demonstrated here represents a broadly conserved mechanism in glioblastoma biology.

In summary, our study reveals NS as a novel MELK substrate and define a previously uncharacterized MELK–NS signaling axis that contributes to glioblastoma progression. This mechanistic understanding supports MELK as a promising therapeutic target and opens new avenues for exploring kinase-mediated regulation of nucleolar proteins in high-grade gliomas [47-50].

## Supplemental Materials

Supplementary data for this paper are available on-line only at http://jmb.or.kr.
