# Supplementary material for: Discovery of the MELK–Nucleostemin Axis in Glioblastoma: Implications for p53 Regulation and Tumor Progression
Source: J Microbiol Biotechnol. 2026 Jan 21;36:e2510047. doi: 10.4014/jmb.2510.10047 (PMC12868952; doi:10.4014/jmb.2510.10047)
Supplement: Supplementary file 1 [file jmb-36-e2510047-supple.pdf]

J. Microbiol. Biotechnol. 2026. 36: e2510047
https://doi.org/10.4014/jmb.2510.10047

Supplementary Figures

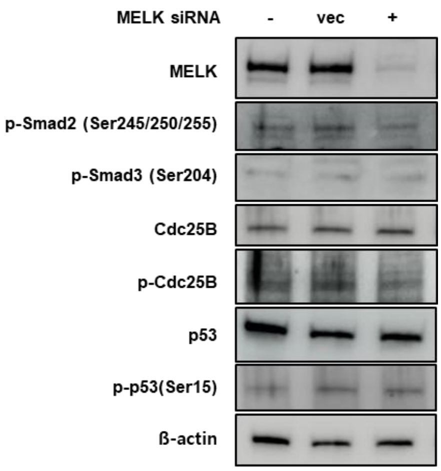

Fig. S1. Expression of various well-known MELK substrate by MELK reduction using MELK siRNA.

Phosphorylation of Cdc25B, Smad2, Smad3 and p53, well known MELK substrate, was examined by western blotting assay. When the cells were transfected with 400 pmole MELK siRNA for 48 hours, phosphorylation of these proteins did was not significantly changed.

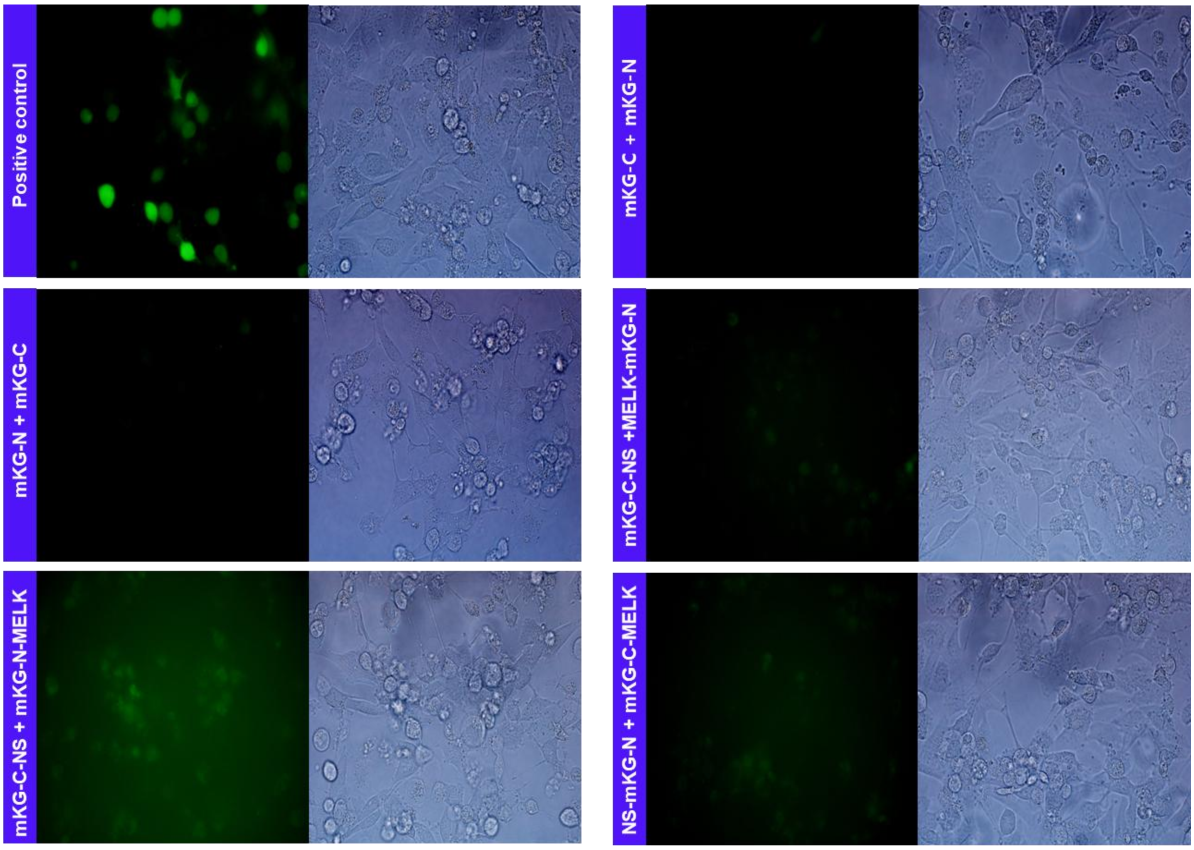

Fig. S2. Association between MELK and nucleostemin proteins on U87MG cells. The U87MG cells were co-transfected cloning vectors inserted MELK and NS cDNA using CoralHue Fluo-chase kit for 48 h. Several fluoroscence expression cotransfected cloning vectors inserted MELK and NS relatively increased on U87MG cells. Especially the cells cotransfected both mKG-C-NS and mKG-N-MELK observed remarkable fluoroscence signal. (X 400)

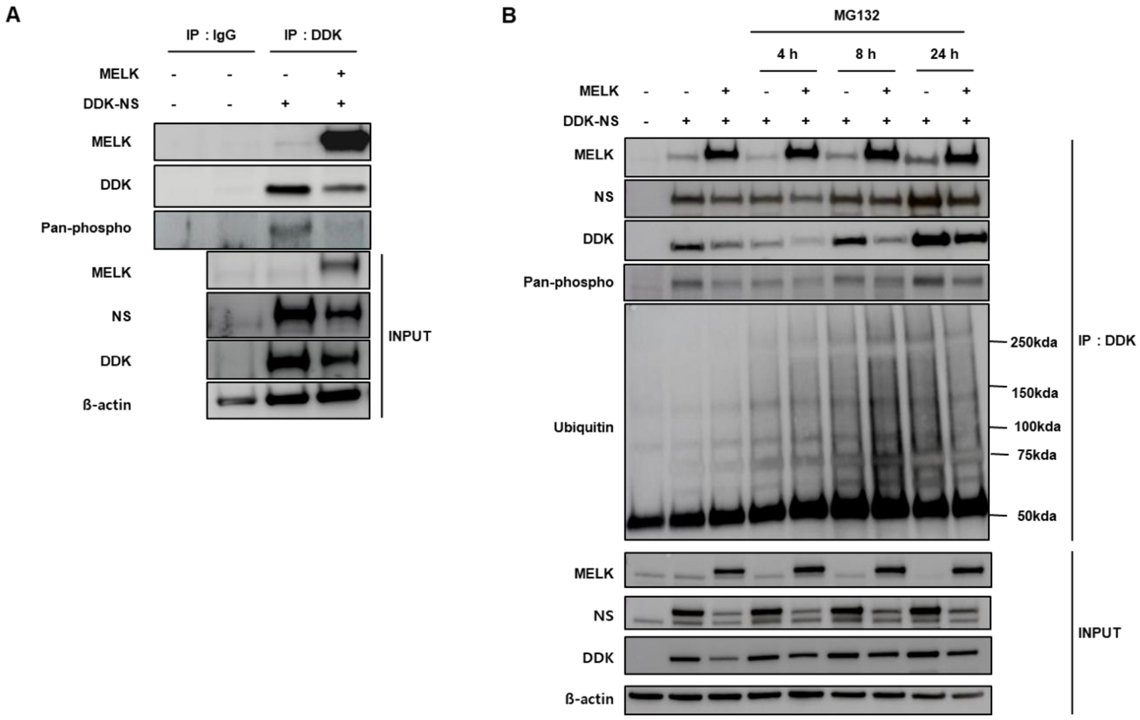

Fig. S3. Decrease of NS level by up-regulation of MELK.

(A) DDK-NS was cotransfected with MELK or vector alone into U87MG cells. DDK-NS proteins were purified using IgG isotype control or DDK antibody, and complex between MELK and NS was confirmed by western blotting assay using MELK and NS antibody. NS was significantly decreased by exogenous induction of MELK on U87MG cells. **(B)** Ubiquitination and recovery of NS by treatment with MG132 in a time-dependent manner.
